# Electronic and optical properties of halogen-substituted LaBi_2_Cl_1−*y*_X_*y*_O_4_: a promising candidate for energy-efficient devices

**DOI:** 10.1039/d5ra06049d

**Published:** 2025-09-29

**Authors:** Dipendra Prasad Kalauni, Kedar Nath Jaiswal, Sarita Lawaju, Krishna Bahadur Rai, Ram Jeewan Yadav, Akkal Dev Mishra, Madhav Prasad Ghimire

**Affiliations:** a Central Department of Physics, Tribhuvan University Kirtipur 44613 Kathmandu Nepal madhav.ghimire@cdp.tu.edu.np; b Department of Chemistry, Prithvi Narayan Campus, Tribhuvan University Pokhara Nepal; c Central Department of Chemistry, Tribhuvan University Kirtipur 44613 Kathmandu Nepal; d Leibniz IFW Dresden Helmholtzstr. 20 01069 Dresden Germany

## Abstract

Layered bismuth oxyhalides exhibit significant promise for photocatalytic and optoelectronic applications owing to their diverse functional characteristics. Here, we report the structural stability and electronic and optical properties of LaBi_2_ClO_4_ and its halogen-substituted derivatives LaBi_2_Cl_1−*y*_X_*y*_O_4_ (X = Br, I; *y* = 0.25, 0.5, 0.75) by means of a density functional theory approach. Both pristine and halogen-substituted compounds are thermodynamically stable, as evidenced by their negative formation energies, and the pristine phase satisfies the mechanical stability criteria. LaBi_2_ClO_4_ is identified as an indirect band gap semiconductor based on electronic structure calculations, with gap values between 1.18 eV (GGA) and 1.98 eV (mBJ). The dominant contributions near the valence and conduction band edges originate from O-2p and Bi-6p electronic states, respectively. Halogen substitution systematically narrows the band gap, with iodine doping inducing the most pronounced reduction, attributed to enhanced lattice distortions and modified orbital hybridization. Optical properties, computed *via* complex dielectric functions, exhibit pronounced anisotropy and tunability; doping shifts absorption edges toward lower photon energies and increases dielectric constants and refractive indices. These tunable optical responses lead to enhanced optical conductivity and reflectivity across the visible to ultraviolet spectrum, underscoring halogen-doped LaBi_2_ClO_4_ as a versatile platform for next-generation optoelectronic devices and photocatalysts.

## Abstract 2

Layered bismuth oxyhalides exhibit significant promise for photocatalytic and optoelectronic applications owing to their diverse functional characteristics.

## Graphical abstract

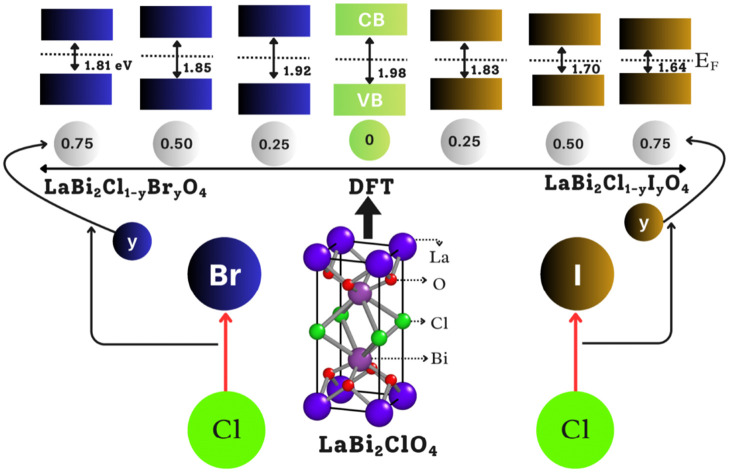

## 1 Introduction

The development of semiconducting materials with tunable optical and electronic properties is critical for advancing next-generation technologies in renewable energy conversion,^1^ environmental remediation,^2^ and optoelectronic applications.^3,4^ In particular, materials capable of efficiently absorbing visible light, generating charge carriers, and facilitating redox reactions are central to addressing challenges in photocatalysis,^5,6^ solar energy harvesting,^7^ and pollutant degradation.^6^ Layered compounds have garnered significant attention in this context due to their intrinsic anisotropy, internal electric fields, and structural flexibility, which promote effective charge separation and enhance light–matter interactions.^8^ Among these, layered oxyhalides stand out as promising candidates, combining oxide–halide frameworks with visible-light responsiveness and offering rich structural and chemical tunability.^9^ Bismuthoxyhalides (BiOX, X = Cl, Br, I) are frontier materials with layered structures, visible light responsiveness, and high charge-separation efficiency.^10,11^ Recent findings reveal outstanding performances in degrading antibiotics (*e.g.*, BiOCl) and herbicides (*e.g.* BiOBr), but electron–hole recombination is still a problem.^12,13^ Semiconductor photocatalysis began in 1972 with the demonstration of water splitting using UV-irradiated TiO_2_ (ref. 14). Since then, the field has grown rapidly, with major advances in efficient photocatalysts. Various semiconductors, including TiO_2_ (ref. 15), ZnO,^16^ ZrO_2_ (ref. 17), SnO_2_ (ref. 18) and Fe_2_O_3_ (ref. 19), have been used as photocatalysts. Among these semiconductors, titanium dioxide (TiO_2_) has emerged as a prototypical n-type material, widely studied for its photocatalytic performance and electronic applications.^14,20^ Metal oxides, including TiO_2_, are vital for environmental cleanup as they create electron–hole pairs when exposed to adequate light energy.^21^ However, while TiO_2_ is widely used in photocatalysis, it only works under UV light, which limits its effectiveness. In contrast, bismuth oxyhalides have an adjustable bandgap ranging from 3.3 eV to 1.8 eV for making them far more efficient at harnessing visible light for photocatalytic reactions.^22,23^ BiOX (X = F, Cl, Br, I) has a layered structure with alternating [Bi_2_O_2_]^2+^ positive layers and negatively charged halogen layers, generating an internal electric field. This promotes efficient electron–hole separation, enhancing photocatalytic performance in light-driven reactions.^24,25^ The band gap of bismuth oxyhalides can be modulated through multiple strategies, such as doping-induced band engineering, noble metal deposition, co-doping, and forming heterojunctions with narrow bandgap semiconductors, as widely documented in studies.^26^ Bi_2_REO_4_X (RE = Y, La–Lu; X = Cl, Br, I) forms a layered crystal structure, representing a combination of oxide and halide-based structural units with similarity in structure with BiOX. Fluorite-like [RE_2_O_2_] layers alternate with single/double halide sheets in these intergrowths.^27–29^ Visible-light-responsive BiOCl_*x*_Br_1−*x*_, BiOCl_*x*_I_1−*x*_, and BiOBr_*x*_I_1−*x*_ with tunable *E*_g_, VB, CB, and surface area (*via x* variation) show strong oxidation ability, making them effective photocatalysts for organic pollutant degradation.^30,31^ Previous studies have shown that the band gap of Bi_2_REO_4_X (RE = Y, La–Lu) compounds can be tuned *via* doping, for instance in Bi_2_Y_1−*x*_M_*x*_O_4_Cl (M = Fe, Ca, Zr).^32,33^ However, the structural and optoelectronic properties of the layered bismuth oxyhalide LaBi_2_ClO_4_, as well as the effects of halogen substitution at the Cl site with Br and I remain unexplored. In this work, we use density functional theory (DFT) to investigate the structural, mechanical, electronic and optoelectronic properties of LaBi_2_ClO_4_, and study the composition-dependent optoelectronic changes induced by Br and I substitution at varying concentrations. These insights extend halogen-tuning strategies to La-based oxyhalides and highlight their potential for optoelectronic applications.

## 2 Computational details

We carried out first-principles density functional theory (DFT) calculations to explore the structural, electronic, and optical properties of LaBi_2_ClO_4_ and its halogen-substituted variants LaBi_2_Cl_1−*y*_Br_*y*_O_4_ and LaBi_2_Cl_1−*y*_I_*y*_O_4_ at doping levels of *y* = 0.25, 0.5, and 0.75. Two computational tools were employed: the Full-Potential Local-Orbital (FPLO) code (v22.00-62)^34^ and the Quantum ESPRESSO (QE v7.2) package.^35^ In FPLO, the energy and charge convergence thresholds were chosen as 10^−8^ Hartree and 10^−6^|*e*|, respectively. Structural optimization involved *k*-point convergence testing followed by lattice parameter optimization. A 12 × 12 × 10 *k*-point mesh was used for electronic structure calculations, while a denser 25 × 25 × 25 *k*-mesh was employed for optical properties. The generalized gradient approximation (GGA) based on the PBE scheme^36^ served as the exchange-correlation functional, and the modified Becke–Johnson (mBJ) potential^37^ was employed to enhance band gap predictions. DFT provides reliable insights into electronic structures and related material properties, as demonstrated in previous studies.^38,39^ To simulate Br and I doping at the Cl site, supercells were constructed. The doped supercells retained the tetragonal crystal symmetry and the original space group. For *y* = 0.25 and *y* = 0.75, 2 × 2 × 1 supercells containing four Cl atoms were used, enabling one- and three-site substitutions, respectively. For *y* = 0.5, a more computationally efficient 1 × 1 × 2 supercell with two Cl atoms was used, allowing 50% substitution while maintaining structural consistency. In all cases, multiple possible substitutional configurations were considered, and the ground-state energies were evaluated to identify the energetically most favorable doping arrangement, which was then used for further analysis. The mechanical properties were assessed using the quasi-harmonic approximation (QHA),^40^ as incorporated in the thermo_pw code within the Quantum ESPRESSO package. The simulations employed a plane-wave basis set, applying cutoff values of 800 Ry for charge density and 80 Ry for kinetic energy. A stringent total energy convergence threshold of 10^−8^ Ry was applied to ensure computational accuracy.

## 3 Results and discussion

### 3.1 Structural stability

**Fig. 1:**
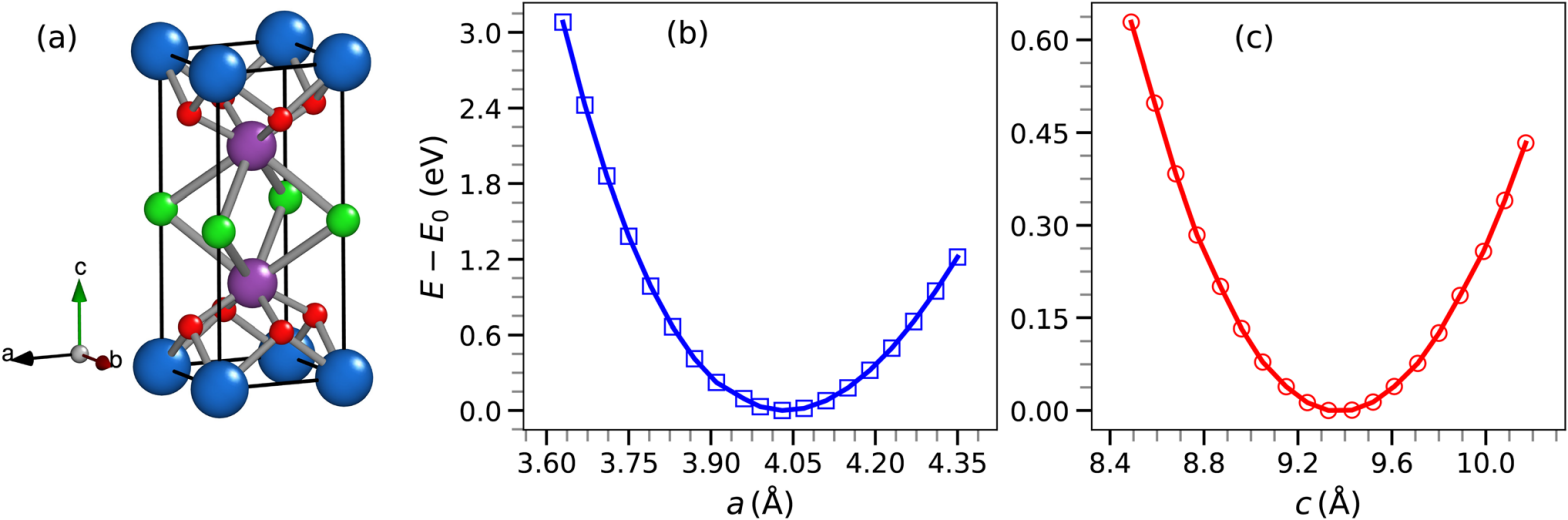
(a) Tetragonal atomic arrangement of LaBi_2_ClO_4_, where blue, violet, green, and red spheres represent La, Bi, Cl, and O atoms, respectively. (b) Relative total energy with respect to lattice parameter *a* (in Å). (c) Relative total energy, (*E* − *E*_0_), with respect to the lattice parameter *c* (in Å). The curves in panels (b) and (c) are fitted using Murnaghan's equation of state.

Bismuth lanthanum oxychloride, LaBi_2_ClO_4_, forms a tetragonal crystal structure belonging to the space group *P*4/*mmm* (no. 123). A primitive unit cell, visualized using XFPLO as implemented in the FPLO package, is shown in Fig. 1(a). The atomic Wyckoff sites within the cell include: La located at (0.0, 0.0, 0.0), Bi at (−0.5, −0.5, 0.278377), Cl at (0.0, 0.0, −0.5), and O at (0.0, −0.5, 0.161656). To assess the structural stability of LaBi_2_ClO_4_, we performed a full structural relaxation by minimizing the total energy relative to the lattice parameters *a* and *c* using DFT within the GGA framework. The total energy difference *E* − *E*_0_ (eV) was evaluated with respect to the lattice constants *a* and *c*, as illustrated in Fig. 1(b) and (c), respectively. Here, *E*_0_ represents the minimum total energy corresponding to the equilibrium configuration. The lattice parameters were optimized by fitting the calculated total energy data to Murnaghan's equation of state,^41^ a commonly used method for describing how the energy of crystalline solids changes with volume. The resulting equilibrium lattice constants for LaBi_2_ClO_4_ are: *a* = *b* = 4.03 Å and *c* = 9.33 Å. These values show good consistency with, though slightly larger than, those reported within the Open Quantum Materials Database (OQMD), which provides *a* = *b* = 3.964 Å and *c* = 9.064 Å.^42^ A comparative summary of the optimized lattice parameters obtained from our calculations, alongside the values reported in the OQMD database and those derived from the 2 × 2 × 1 and 1 × 1 × 2 supercells, are presented in Table 1.

**Table 1:** Optimized lattice constants *a* = *b* and *c* (in Å) for LaBi_2_ClO_4_ obtained from our DFT calculations, compared with OQMD data^42^ and results from 2 × 2 × 1 and 1 × 1 × 2 supercell configurations

Compound	*a* = *b*	*c*	*a*_ref_ = *b*_ref_	*c*_ref_	*a*_2×2×1_ = *b*_2×2×1_	*c*_2×2×1_	*a*_1×1×2_ = *b*_1×1×2_	*c*_1×1×2_
LaBi_2_ClO_4_	4.03	9.33	3.964	9.064	8.06	9.20	4.03	18.66

To evaluate the thermodynamic stability, we calculated the formation energy (*E*_f_) of the parent compound LaBi_2_ClO_4_, obtaining a value of −1.82 eV per atom. The negative formation energy indicates that the compound is thermodynamically stable relative to its elemental constituents. This result compares well with the value of −2.26 eV per atom reported in the OQMD.^42^ The slight discrepancy may arise from differences in computational parameters or exchange–correlation functionals. Upon halogen substitution at the Cl site, the formation energies of the Br-doped compounds LaBi_2_Cl_1−*y*_Br_*y*_O_4_ are found to be −1.81, −1.80, and −1.79 eV per atom for doping levels *y* of 0.25, 0.50, and 0.75, respectively. In comparison, I-doped counterparts LaBi_2_Cl_1−*y*_I_*y*_O_4_ exhibit slightly less negative formation energies of −1.79, −1.76, and −1.73 eV per atom for the same doping levels. These results indicate that all compounds remain thermodynamically stable upon doping, with Br substitution yielding slightly more favorable formation energies than I substitution.

### 3.2 Mechanical properties

The elastic properties of LaBi_2_ClO_4_ were systematically investigated to evaluate its mechanical behavior and stability. The independent elastic constants *C*_*ij*_, derived from the generalized Hooke's law,^43^ serve as fundamental parameters linking atomic bonding characteristics to the macroscopic mechanical response of the material. For the tetragonal phase (space group *P*4/*mmm*) of LaBi_2_ClO_4_, six distinct elastic constants—*C*_11_, *C*_12_, *C*_13_, *C*_33_, *C*_44_, and *C*_66_—were calculated using the QHA method and are shown in Table 2.

**Table 2:** Calculated elastic constants (*C*_*ij*_ in GPa), Young's modulus (*Y* in GPa), shear modulus (*G* in GPa), bulk modulus (*B* in GPa), Pugh's ratio (*B*/*G*), its inverse (*G*/*B*), and Poisson's ratio (*ν*) for the layered bismuth oxyhalide LaBi_2_ClO_4_

Material	*C*_11_	*C*_12_	*C*_13_	*C*_33_	*C*_44_	*C*_66_	*Y*	*G*	*B*	*B*/*G*	*G*/*B*	*ν*
LaBi_2_ClO_4_	146.64	64.04	25.41	78.85	10.41	63.79	69.82	26.72	62.32	2.33	0.43	0.31

Mechanical properties of a material provide critical insights into its response to external stresses and are key indicators of its structural stability. The mechanical stability of such crystals is governed according to the Born criteria. In the case of a tetragonal system classified as type I,^44^ the structure is mechanically stable if the following conditions are satisfied: *C*_11_ > |*C*_12_|, *C*_33_ > 0, *C*_44_ > 0, *C*_66_ > 0, and 2*C*_13_^2^ < *C*_33_(*C*_11_ + *C*_12_). The computed elastic constants for LaBi_2_ClO_4_, listed in Table 2, satisfy all of these conditions, confirming that the compound is mechanically stable. The elastic response of LaBi_2_ClO_4_ was further characterized through the evaluation of macroscopic mechanical moduli derived from the computed elastic constants. The bulk modulus (*B* = 62.32 GPa) reflects the material's resistance to isotropic compression, indicating moderate incompressibility under hydrostatic pressure. The shear modulus (*G* = 26.72 GPa), which governs the material's resistance to shape deformation under shear stress, is relatively low, consistent with the layered structure of the compound.^45^ Young's modulus (*Y* = 69.82 GPa), which quantifies stiffness under uniaxial strain,^46,47^ also supports the conclusion that LaBi_2_ClO_4_ exhibits moderate elastic rigidity. The Pugh's ratio^48^ (*B*/*G* = 2.33) significantly exceeds the empirical ductile–brittle threshold of 1.75, indicating pronounced ductility. This is further corroborated by its inverse (*G*/*B* = 0.43), which lies well below unity, reaffirming the ductile mechanical character. The calculated Poisson's ratio (*ν* = 0.31) falls within the typical range for ionic solids, suggesting an intermediate bonding character with a dominant ionic contribution.^49^ Additionally, a Poisson's ratio close to 0.5 implies low volume change under stress; thus, the observed value suggests that LaBi_2_ClO_4_ possesses relatively low compressibility and good mechanical stability. These findings—moderate stiffness, high ductility, and ionic bonding character—are consistent with previously reported mechanical behavior of structurally analogous layered bismuth oxyhalides, such as Bi_2_LaO_4_I.^50^

### 3.3 Electronic properties

In order to determine the ground-state properties of LaBi_2_ClO_4_, both nonmagnetic and spin-polarized configurations were investigated. The total energies of the two configurations were found to be identical within the convergence threshold, and the spin-polarized calculations yielded zero magnetic moments, indicating a nonmagnetic ground state. To investigate the electronic configuration of LaBi_2_ClO_4_, density functional theory calculations were carried out employing both GGA and mBJ exchange potentials. The overall features of the electronic band structure remain similar in both approaches, except for a significant increase in the band gap observed with the mBJ functional. We focus on mBJ calculations for the electronic band structure because the GGA approach underestimates band gaps,^51,52^ whereas mBJ closely reproduces experimental trends, as shown by the agreement between measured and calculated band gaps in this class of oxyhalides,^32^ demonstrating its reliability for accurately predicting their electronic properties. For comparison, the GGA results are provided in the SI, while this section emphasizes the findings obtained using mBJ, with occasional references to GGA where relevant.

**Fig. 2:**
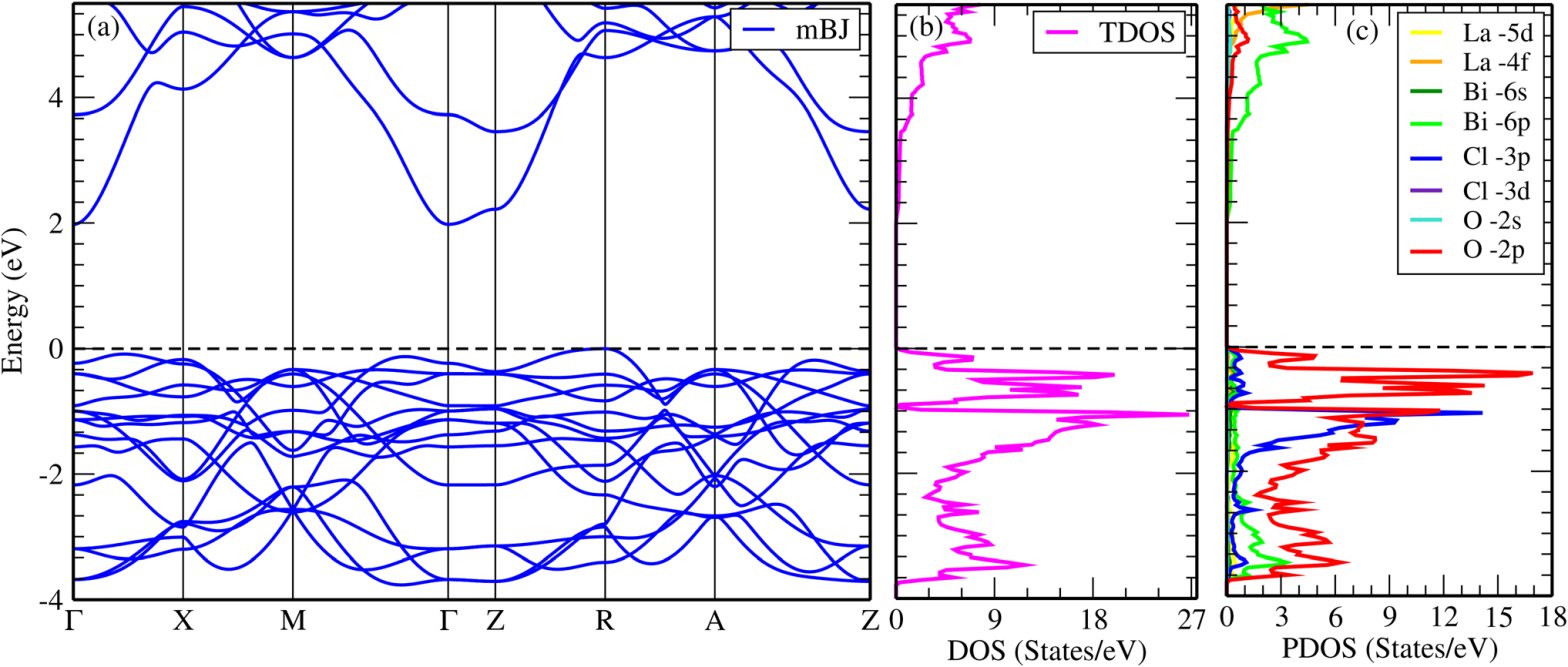
Calculated electronic properties of LaBi_2_ClO_4_ using the mBJ potential: (a) band structure, (b) total density of states (DOS), and (c) projected density of states (PDOS). Dashed horizontal lines in all plots denote the Fermi level (*E*_F_).

Fig. 2(a) illustrates the electronic band structure for LaBi_2_ClO_4_ obtained using the mBJ approach. The valence band maximum (VBM) occurs at the high-symmetry point *R*, while the conduction band minimum (CBM) appears at the *Γ* point, confirming that LaBi_2_ClO_4_ exhibits an indirect band gap.^53^ Such an indirect transition is advantageous for reducing the recombination probability of photoinduced carriers, as the photoexcited electrons must traverse a finite distance in *k*-space before recombination occurs.^54,55^ Using the GGA method, the indirect band gap is calculated as 1.18 eV (see SI: Fig. S1(a)), whereas the mBJ approach yields a significantly larger value of 1.98 eV, consistent with the well-known band gap underestimation tendency of GGA.^51^ For LaBi_2_ClO_4_ to be effective in photoelectronic applications, both the size and the nature of its band gap are of great importance. The band gap size influences how the material interacts with sunlight across the spectrum, while the gap type governs charge carrier behavior. Materials with a direct band gap typically absorb photons more efficiently, whereas those with an indirect band gap tend to promote better separation of photoexcited electrons and holes, thereby reducing their likelihood of recombination.^56^ This behavior aligns well with previously reported findings on structurally related layered bismuth oxyhalides, including Bi_2_LaO_4_I^50^ and Bi_2_YO_4_Cl,^32^ which shows comparable electronic and crystallographic features.

Fig. 2(b) and (c) depict the total and partial density of states (DOS and PDOS) of LaBi_2_ClO_4_, respectively. As observed in the total DOS, a significant band gap of around 1.18 eV appears using the GGA approach, while a larger gap of 1.98 eV is found with the mBJ functional, consistent with the results of the electronic band structure. The orbital-resolved PDOS shown in Fig. 2(c) (see SI: Fig. S1(c)) reveals that the valence band states close to the Fermi level (*E*_F_) are primarily derived from O-2p, Cl-3p, and Bi-6s orbitals, whereas the conduction band region largely consists of Bi-6p, La-4f states, and La-5d contributions. La-5d, La-4f, and Bi-6s states contribute minimally to the valence band, while Cl-3d, Cl-3p, and O-2s orbitals exhibit negligible presence in the conduction band region. There is a marked hybridization between the Cl-3p and O-2p orbitals in the valence band, revealing substantial p–p interaction in this area. In contrast, a modest degree of hybridization is present between Cl-3p and Bi-6p orbitals within the conduction band. Furthermore, Bi-6p and La-5d orbitals contribute predominantly to the conduction band states, particularly at higher energies above the Fermi level, although their mutual hybridization remains moderate. These orbital characteristics give rise to a wide band gap and a favorable electronic structure, rendering LaBi_2_ClO_4_ a promising material for optoelectronic applications such as photocatalysis, photodetection, and light-emitting diodes (LEDs).^4^

**Fig. 3:**
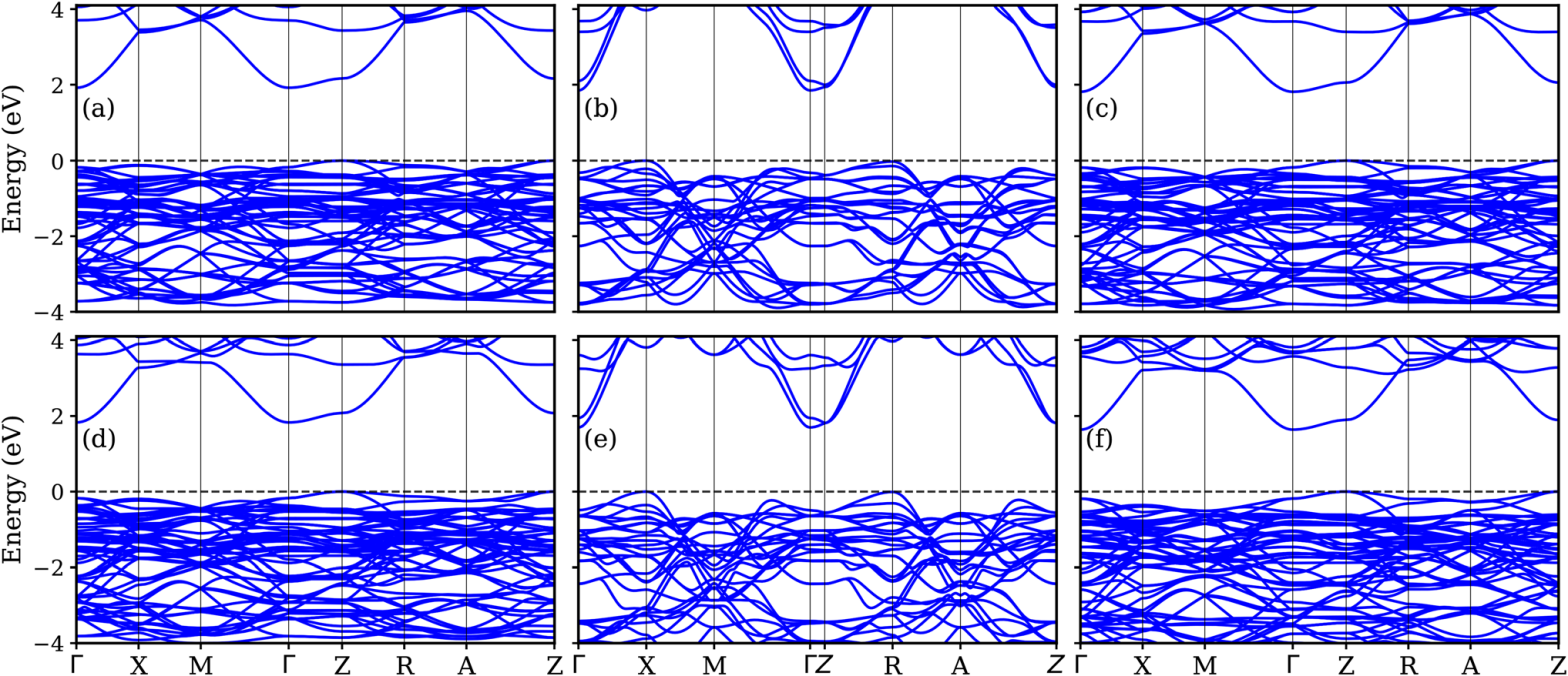
Electronic band structures of LaBi_2_ClO_4_ with partial substitution of Cl by Br and I at various doping concentrations, calculated using the mBJ potential: (a) LaBi_2_Cl_0.75_Br_0.25_O_4_, (b) LaBi_2_Cl_0.5_Br_0.5_O_4_, (c) LaBi_2_Cl_0.25_Br_0.75_O_4_, (d) LaBi_2_Cl_0.75_I_0.25_O_4_, (e) LaBi_2_Cl_0.5_I_0.5_O_4_, and (f) LaBi_2_Cl_0.25_I_0.75_O_4_.

**Fig. 4:**
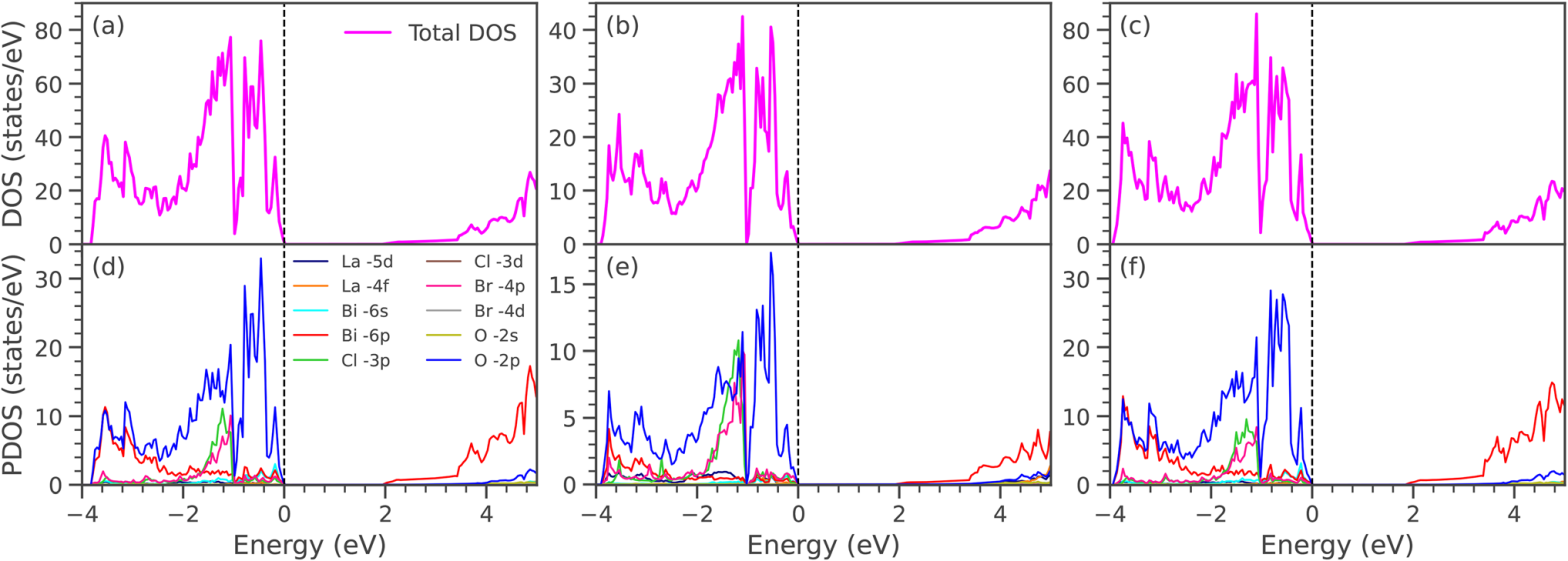
Total and partial DOS of (a and d) LaBi_2_Cl_0.75_Br_0.25_O_4_, (b and e) LaBi_2_Cl_0.5_Br_0.5_O_4_, (c and f) LaBi_2_Cl_0.25_Br_0.75_O_4_, calculated using the mBJ potential.

**Fig. 5:**
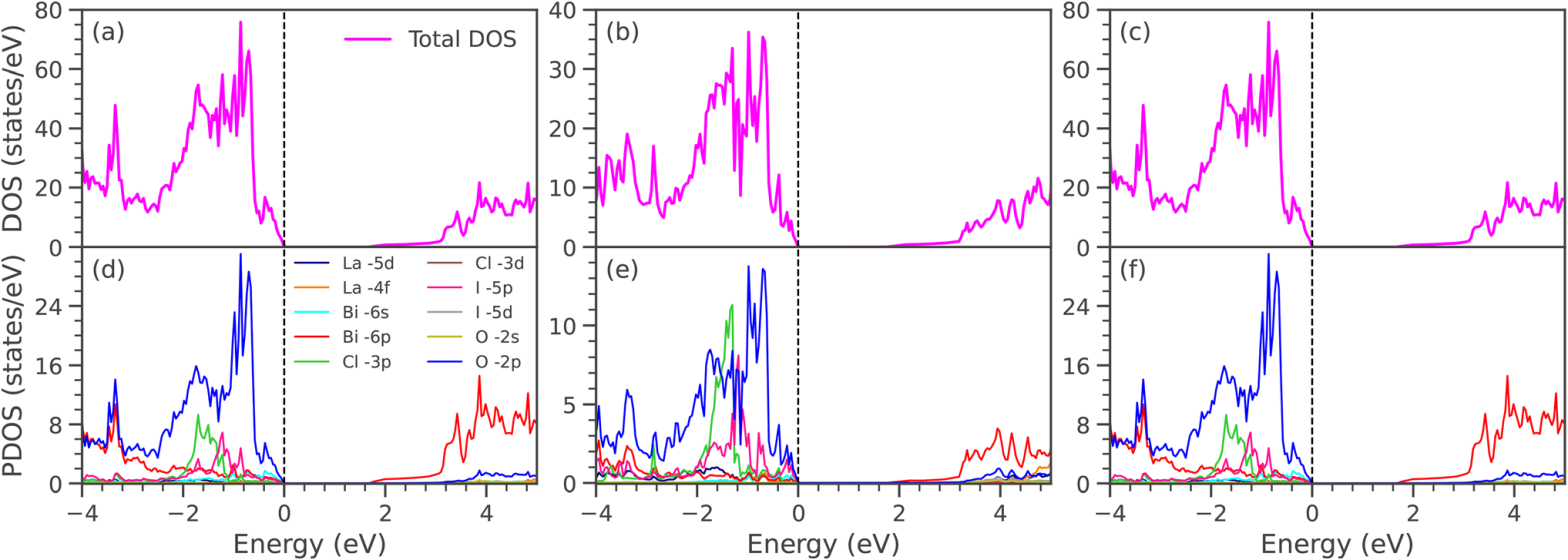
Total and partial DOS of (a and d) LaBi_2_Cl_0.75_I_0.25_O_4_, (b and e) LaBi_2_Cl_0.5_I_0.5_O_4_, and (c and f) LaBi_2_Cl_0.25_I_0.75_O_4_ calculated using the mBJ potential.

Fig. 3(a–c) displays the electronic band structures of LaBi_2_Cl_1−*y*_Br_*y*_O_4_, while Fig. 3(d–f) shows those of LaBi_2_Cl_1−*y*_I_*y*_O_4_ for doping levels *y* = 0.25, 0.50, and 0.75, where Cl atoms are partially substituted by Br and I, respectively. To model 25% doping, one Cl atom out of four was substituted in a 2 × 2 × 1 supercell. For 50% doping, one Cl atom out of two was replaced in a 1 × 1 × 2 supercell. In the case of 75% doping, three Cl atoms out of four were substituted in a 2 × 2 × 1 supercell. In all doped configurations, the CBM and VBM appear at different high-symmetry points, confirming that the systems remain indirect band gap semiconductors, consistent with pristine LaBi_2_ClO_4_ (band gap: 1.18 eV, GGA; 1.98 eV, mBJ; VBM at *R*, CBM at *Γ*). At *y* = 0.25, replacing Cl with Br results in an indirect band gap of 1.13 eV (GGA) and 1.92 eV (mBJ). A similar substitution with iodine yields slightly lower values of 1.07 eV (GGA) and 1.83 eV (mBJ). These values show a decrease compared to pristine LaBi_2_ClO_4_, indicating the pronounced influence of heavier halogen atoms on the material's electronic structure. The initial reduction in band gap arises from the larger atomic radii of Br and I,^57–59^ which expand the lattice and perturb the crystal field. With increased substitution (*y* = 0.50), this trend continues: Br doping yields band gaps of 1.08 eV (GGA) and 1.92 eV (mBJ), while I doping further lowers the gap to 1.02 eV (GGA) and 1.70 eV (mBJ). At *y* = 0.75, the Br-substituted compound shows values of 1.05 eV (GGA) and 1.85 eV (mBJ), whereas I doping reduces the band gap to 0.96 eV (GGA) and 1.64 eV (mBJ). These findings reveal a monotonic decrease in the indirect band gap with progressive halogen substitution. This effect is attributed to the increased ionic radii of Br and I, which induce local lattice distortions and modify the bonding environment. As a result, these structural changes affect orbital hybridization and shift the energy positions of the valence and conduction bands. Such tunability of the electronic properties underscores the potential of halogen-substituted layered oxyhalides for optoelectronic^60^ and photocatalytic applications,^9,32^ where precise control over the band gap is essential. Fig. 4(a–f) shows the DOS and PDOS of LaBi_2_Cl_1–*y*_Br_*y*_O_4_ at substitution levels of *y* = 0.25, 0.50, and 0.75. The total DOS plots (Fig. 4(a–c)) reveal a gradual narrowing of the band gap with increasing Br concentration, decreasing from approximately 1.13 to 1.05 eV based on the GGA results (see SI: Fig. S3(a–c)). The modified Becke–Johnson (mBJ) potential, in contrast, yields higher band gap values in the range of 1.92 to 1.81 eV, which align well with the electronic band structures illustrated in Fig. 3(a–c). The PDOS plots (Fig. 4(d–f)) reveal that the states in the vicinity of the Fermi energy (*E*_F_) within the valence band region are predominantly derived from O-2p and Cl-3p orbitals, while Br-4p contributions become more pronounced as doping increases. The CBM is primarily composed of Bi-6p states. Lesser yet notable contributions in the conduction region come from La-5d, La-4f, and O-2s orbitals, with moderate involvement of Bi-6s and Bi-6p states in the valence region. Orbitals such as La-5d, La-4f, and Bi-6s (valence) and Cl-3d, Cl-3p, and O-2s (conduction) exhibit minimal involvement. Distinct hybridization among Cl-3p, O-2p, and Br-4p orbitals is evident in the valence band, alongside intermediate hybridization between Cl-3p and Bi-6p. Additionally, weak interactions are identified between Bi-6s and O-2p, and moderate hybridization occurs between O-2s and Bi-6p in the conduction band region. Fig. 5(a–f) displays the total and orbital-resolved DOS for LaBi_2_Cl_1–*y*_I_*y*_O_4_ at the same doping levels. As depicted in the total DOS (Fig. 5(a–c)) a progressive narrowing of the GGA band gap occurs from 1.07 to 0.96 eV with increasing I content (see SI: Fig. S3(d–f)). The mBJ-calculated gaps span a higher range, decreasing slightly from 1.83 to 1.64 eV, in line with the corresponding band structures (Fig. 3(d–f)). According to the PDOS (Fig. 5(d–f)), the valence states near *E*_F_ primarily originate from O-2p and Cl-3p orbitals, while I-5p orbitals exhibit growing intensity with increasing substitution. Bi-6p states remain the dominant contributors at the CBM throughout the doping range. Furthermore, La-5d, La-4f, and O-2s orbitals make modest contributions in the conduction band, while Bi-6s and Bi-6p are visible in the valence region. Orbitals such as La-5d, La-4f, and Bi-6s (valence), along with Cl-3d, Cl-3p, and O-2s (conduction), have negligible impact. Prominent hybridization is noted among Cl-3p, O-2p, and I-5p orbitals in the valence region, complemented by moderate Cl-3p–Bi-6p interactions. Weak coupling between Bi-6s and O-2p, as well as moderate O-2s–Bi-6p hybridization in the conduction band, is also observed. Compared with co-doping and heterostructure-based strategies, halogen substitution offers a simpler and more reproducible approach for band-gap engineering. Co-doping, such as Al–Co in TiO_2_ (ref. 61) or La–N in SrTiO_3_,^62^ achieves comparable reductions in the electronic gap (3.11 eV → 1.97 eV and 2.20 eV → 1.02 eV) but requires multi-dopant incorporation, which can introduce defect states. Heterostructures, including ZnO/BSe^63^ and HfS_2_/MoTe_2_,^64^ enable precise band alignment but demand complex interface engineering. In contrast, single-site halogen doping is straightforward, though very high substitution levels may induce lattice strain or defects. This tunable strategy positions LaBi_2_ClO_4_ as a promising candidate for optoelectronic and photocatalytic applications.

### 3.4 Optical properties

**Fig. 6:**
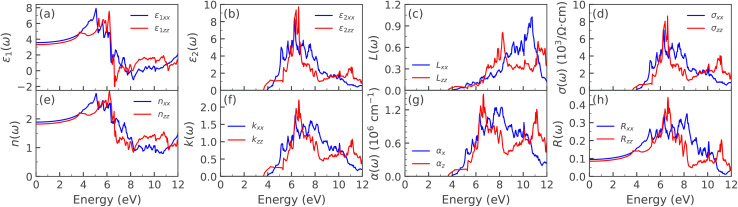
Calculated optical properties of LaBi_2_ClO_4_ as functions of photon energy using the mBJ exchange potential. Panels show: (a) real *ε*_1_(*ω*) and (b) imaginary *ε*_2_(*ω*) parts of the dielectric function; (c) energy loss function *L*(*ω*); (d) optical conductivity *σ*(*ω*); (e) refractive index *n*(*ω*); (f) extinction coefficient *k*(*ω*); (g) absorption coefficient *α*(*ω*); and (h) reflectivity *R*(*ω*). The subscripts *xx* and *zz* denote the polarization directions along the crystallographic *x*- and *z*-axes, respectively.

Fig. 6(a–h) illustrates the optical properties of LaBi_2_ClO_4_ computed using the mBJ exchange potential. These include (a) the real and (b) imaginary parts of the dielectric function, (c) energy loss function, (d) optical conductivity, (e) refractive index, (f) extinction coefficient, (g) absorption coefficient, and (h) reflectivity. The *xx* and *zz* components refer to the polarization along the *x*- and *z*-axes, respectively. For comparison, the corresponding optical properties were also calculated using the GGA functional. The results exhibit the same overall behavior as mBJ, with only slight differences in magnitude (see SI for details).

The complex dielectric function is a fundamental quantity used to characterize the optical response of materials.^65–67^ It describes how a material interacts with incident electromagnetic radiation and governs various frequency-dependent optical properties. The real part of the dielectric function, *ε*_1_(*ω*), reflects the material's polarization response and dispersion behavior, while the imaginary part, *ε*_2_(*ω*), is directly related to the absorption of electromagnetic radiation and reveals information about interband electronic transitions. As such, the dielectric function serves as a central parameter through which other optical constants can be derived and analyzed. Fig. 6(a) presents the real part of the dielectric function, *ε*_1_(*ω*), in terms of photon energy ranging from 0 to 12 eV. The static dielectric constant is given by *ε*_1_(0) at zero frequency, with calculated values of 3.56 and 3.30 along the crystallographic directions *xx* and *zz*, respectively. The static dielectric function indicates the degree of charge recombination in a material, where a higher value corresponds to a lower recombination rate, thereby playing a crucial role in enhancing the operational effectiveness of optoelectronic devices. When the value moves into the UV spectrum, it begins to decline slowly, causing the real part of the dielectric function to take on negative values within this range.^68^ Between 6.59 and 10.1 eV, the real part of the dielectric function for the compound exhibits negative values. Within this energy range, the dielectric function repeatedly attains negative values due to multiple peaks in the visible and UV regions. With increasing photon energy, *ε*_1_(*ω*) increases, reaching maxima of 7.92 at 5.07 eV (*xx*) and 7.54 at 6.19 eV (*zz*). Beyond these energies, *ε*_1_(*ω*) decreases and turns negative, a behavior commonly linked to interband transitions that align with significant peaks in the dielectric function's imaginary component.

**Fig. 7:**
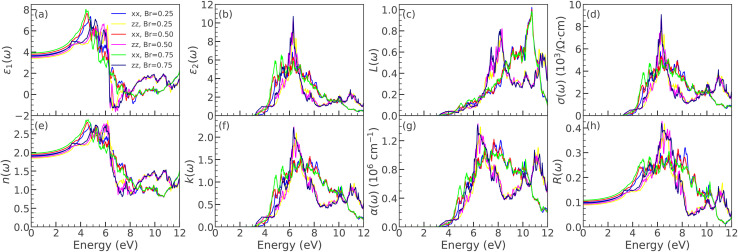
Optical spectra of LaBi_2_Cl_1−*y*_Br_*y*_O_4_ (*y* = 0.25, 0.50, 0.75) using mBJ, showing (a)–(h): *ε*_1_, *ε*_2_, *L*, *σ*, *n*, *k*, *α*, and *R* for *xx* and *zz* polarizations.

**Fig. 8:**
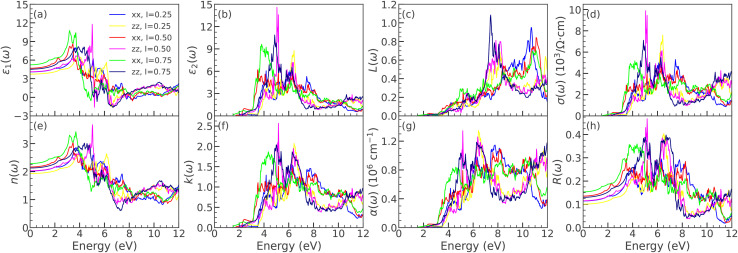
(g) Optical spectra of LaBi_2_Cl_1−*y*_I_*y*_O_4_ (*y* = 0.25, 0.50, 0.75) using mBJ, showing (a)–(h): *ε*_1_, *ε*_2_, *L*, *σ*, *n*, *k*, *α*, and *R* for *xx* and *zz* polarizations.

Variation of the imaginary component of the dielectric function, *ε*_2_(*ω*), with respect to photon energy is depicted in Fig. 6(b). Peaks in *ε*_2_(*ω*) reflect transitions of electrons between the valence and conduction bands and indicate the energy levels at which photons are absorbed. These absorption characteristics reveal details about the compound's interaction with incoming radiation. The optical absorption begins at around 3.65 eV, indicating the optical band gap of LaBi_2_ClO_4_. Notable anisotropy is observed between the *xx* and *zz* polarization directions. Distinct absorption peaks appear at 5.17, 5.71, 6.33, 6.99, and 7.59 eV for the *xx* polarization, and at 5.93, 6.37, and 6.62 eV for the *zz* polarization. These features correspond to interband transitions, primarily from the O 2p valence band to the Bi 6p conduction band, which are consistent with the trends observed in *ε*_1_(*ω*). The energy dissipated by a fast electron passing through the material is characterized by the energy-loss function, *L*(*ω*), as presented in Fig. 6(c).^69^ The peaks in *L*(*ω*) are typically associated with plasmon excitations. A pronounced peak is observed at approximately 10.7 eV along the *xx*-direction, indicating significant energy loss in this polarization. In contrast, the peak along the *zz*-direction appears at a lower energy of around 8.5 eV, highlighting the anisotropic plasmonic response of LaBi_2_ClO_4_. The optical conductivity, *σ*(*ω*), shown in Fig. 6(d), describes the process in which electrons are excited from energy states within the valence band into available states in the conduction band through photon absorption.^69^ It follows a similar profile to that of *ε*_2_(*ω*), as they are related by the expression 
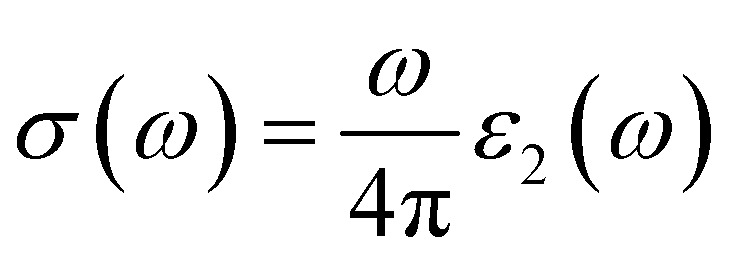
. Several sharp features are observed in the conductivity spectrum, which may arise from interband transitions and plasmonic excitations. The maximum values of *σ*(*ω*) along the *xx* and *zz* polarization directions are approximately 6.9 and 8.6 (× 10^3^ Ω^−1^ cm^−1^), at photon energies of 6.31 and 6.62 eV, respectively. Fig. 6(e) illustrates how the refractive index, *n*(*ω*), of LaBi_2_ClO_4_ varies with photon energy. The refractive index is a fundamental optical parameter that quantifies the reduction in the speed of light within a medium and governs the refraction of light at material interfaces.^70^ Materials with a high refractive index—typically exceeding 1.8—are particularly desirable for optoelectronic and photovoltaic applications^66^ due to their enhanced light–matter interaction capabilities. As evident from Fig. 6(e), LaBi_2_ClO_4_ exhibits a static refractive index of approximately 1.88 and 1.81 along the *xx* and *zz* directions, respectively, at zero photon energy. The refractive index attains peak values of 2.87 in the *xx* direction and 2.96 in the *zz* direction at around 5.13 eV and 6.19 eV photon energies. This relatively high refractive index implies strong photon confinement and an enhanced optical response, properties that are advantageous for applications in solar cells, photodetectors, and light-emitting diodes.^4^ The absorption and scattering behavior of electromagnetic radiation in the material, as a function of photon energy, is described by the extinction coefficient *k*(*ω*).^71^Fig. 6(f) illustrates that LaBi_2_ClO_4_ exhibits a notable rise in *k*(*ω*) near 6.5 eV, reaching a maximum value of approximately 2.25, which indicates strong optical absorption in this energy range. The absorption coefficient quantifies the optical energy loss in a material as light passes through a specific length.^72^ Optical absorption is triggered when the photon frequency coincides with electronic transitions inside the material. The absorption coefficient, *α*(*ω*), varies with photon energy, reflecting the material's ability to absorb light at specific frequencies. When photons possess sufficient energy, electrons are excited from the valence band to the conduction band, resulting in optical absorption. Fig. 6(g) presents the photon-energy-dependent absorption coefficient, *α*(*ω*), of LaBi_2_ClO_4_. A distinct absorption edge appears near ∼3.65 eV, marking the onset of interband transitions. Beyond this threshold, *α*(*ω*) increases significantly, and several pronounced peaks are observed between 5.0 and 11.5 eV, indicating strong optical activity and photon absorption in this energy range. The reflectivity spectrum, *R*(*ω*), is presented in Fig. 6(h). The maximum reflectivity occurs near 6.33 and 6.65 eV, with peak values of 34% and 43% along the *xx*- and *zz*-directions, respectively. The relatively low reflectivity throughout the examined energy range indicates strong optical absorption in LaBi_2_ClO_4_, with minimal energy loss due to reflection, thereby suggesting its potential suitability for optoelectronic applications.^53^ The influence of halide substitution on the optical properties of LaBi_2_ClO_4_ was systematically investigated for Br and I doping at concentrations of *y* = 0.25, 0.50, and 0.75. Fig. 7(a)–(h) and 8(a)–(h) present the frequency-dependent optical parameters of LaBi_2_Cl_1−*y*_Br_*y*_O_4_ and LaBi_2_Cl_1−*y*_I_*y*_O_4_, respectively. Fig. 7(a) and 8(a) present the real part of the dielectric function, *ε*_1_(*ω*). The static dielectric constant, *ε*_1_(0), increases with substitution in both Br- and I-doped systems. Compared to the parent compound (3.56/3.30 along the *xx*/*zz* directions), Br substitution leads to moderate increases, reaching 3.68/3.40 for *y* = 0.25, 3.79/3.50 for *y* = 0.50, and 3.91/3.60 for *y* = 0.75. For I substitution, the increase is more pronounced, with *ε*_1_(0) values of 4.09/3.73 for *y* = 0.25, 4.71/4.15 for *y* = 0.50, and 5.24/4.53 for *y* = 0.75, indicating enhanced polarizability due to the heavier halogen. Additionally, the main peaks in *ε*_1_(*ω*) shift to higher energies with increasing *y*, in contrast to the peak positions at 5.07 eV (*xx*) and 6.19 eV (*zz*) in the undoped material. Fig. 7(b) and 8(b) show the imaginary part, *ε*_2_(*ω*). The optical absorption onset redshifts with doping. In the Br-doped case, it shifts from 3.42 eV (*y* = 0.25) to 2.75 eV (*y* = 0.75); for I doping, a more substantial shift occurs—from 1.93 eV (*y* = 0.25) to 1.47 eV (*y* = 0.75). These features indicate narrowing band gaps and are attributed to interband transitions involving O 2p to Bi 6p states. The shift is more prominent in I-doped compounds, demonstrating stronger modification of the optical band structure. The energy-loss function *L*(*ω*) (Fig. 7(c) and 8(c)) exhibits pronounced plasmon peaks. In the *xx* direction, the plasmon peak remains near 10.7 eV for all Br and I concentrations, comparable to the undoped system. Along the *zz* direction, Br substitution slightly increases the peak energy from 8.17 eV at *y* = 0.25 to 8.31 eV at *y* = 0.50, while it remains relatively unchanged at *y* = 0.75. In contrast, I doping leads to a downward trend in peak positions, decreasing from 8.17 eV at *x* = 0.25 to 7.43 eV at *y* = 0.75, indicating a softened plasmonic response and enhanced anisotropy in dielectric behavior. Fig. 7(d) and 8(d) present the optical conductivity, *σ*(*ω*), in units of Ω^−1^ cm^−1^, for Br- and I-doped LaBi_2_ClO_4_. In Br-doped systems, peak values increase with doping, especially along the *zz* direction: for *y* = 0.25, *σ*(*ω*) reaches 5.7 × 10^3^ (*xx*, at 6.22 eV) and 7.8 × 10^3^ (*zz*, at 6.31 eV); at *y* = 0.50, 5.3 × 10^3^ (*xx*) and 8.9 × 10^3^ (*zz*); and at *y* = 0.75, 5.1 × 10^3^ (*xx*) and 9.1 × 10^3^ (*zz*). Iodine doping follows a non-monotonic trend: at *y* = 0.25, *σ*(*ω*) peaks at 5.4 × 10^3^ (*xx*, 6.21 eV) and 7.6 × 10^3^ (*zz*, 6.43 eV); increases to 4.8 × 10^3^ (*xx*) and 9.9 × 10^3^ (*zz*) at *y* = 0.50; then drops to 7.1 × 10^3^ (*xx*) and 5.2 × 10^3^ (*zz*) at *y* = 0.75. Compared to the parent compound, both dopants enhance *σ*(*ω*)—more significantly along the *zz* direction—with the strongest photoconductivity observed at intermediate concentrations. The refractive index *n*(*ω*), shown in Fig. 7(e) and 8(e), reveals notable changes upon doping. The undoped LaBi_2_ClO_4_ has static refractive indices of 1.88 (*xx*) and 1.81 (*zz*). Upon Br doping, *n*(0) increases gradually relative to the parent: from 1.91/1.84 at *y* = 0.25, to 1.94/1.87 at *y* = 0.50, and 1.98/1.89 at *y* = 0.75. In contrast, I doping leads to a more pronounced increase compared to the parent values, with *n*(0) rising to 2.02/1.93 (*y* = 0.25), 2.48/2.05 (*y* = 0.50), and 2.89/2.13 (*y* = 0.75), indicating significantly enhanced optical confinement. The extinction coefficient *k*(*ω*), depicted in Fig. 7(f) and 8(f), reflects absorption and scattering behavior. For Br doping, *k*(*ω*) shows peaks above 6 eV across all doping levels, with slight redshifts as *y* increases. In contrast, I doping results in more pronounced shifts: 6.22 eV (*y* = 0.25), 5.18 eV (*y* = 0.50), and 4.37 eV (*y* = 0.75), consistent with the band gap reduction observed earlier. Fig. 7(g) and 8(g) display the absorption coefficient *α*(*ω*). In LaBi_2_ClO_4_, absorption begins around 3.65 eV and rises steeply, with strong peaks up to 11.5 eV. Br doping causes a moderate redshift of the absorption edge across *y* = 0.25 to 0.75, while I doping causes a significant redshift with sharper absorption features, especially at *y* = 0.50 and 0.75, signifying stronger interband transitions and enhanced optical activity. Finally, the reflectivity spectra *R*(*ω*) (Fig. 7(h) and 8(h)) reveal moderate changes due to doping. For the parent compound, reflectivity peaks at 34% (*xx*) and 43% (*zz*). Br-doped systems show a slight reduction in the *xx* direction from 32% (*x* = 0.25) to 30% (*y* = 0.75). In I-doped systems, the reflectivity shows stronger modulation, with a notable maximum of 47% in the *zz* direction at *y* = 0.50, indicating tunability of surface optical properties by iodine substitution for potential optoelectronic applications. Given the prominent role of halogen substitution in modulating electronic properties, LaBi_2_ClO_4_ was studied within the broader class of layered bismuth oxyhalides. Experimental studies report indirect band gaps of 2.40 eV for Bi_2_YO_4_Cl, 2.21 eV for Bi_2_EuO_4_Cl, and 1.89 eV for Bi_2_NdO_4_Cl, with DFT closely matching the indirect nature, although GGA underestimates some gaps (*e.g.*, 1.23 eV for Bi_2_NdO_4_Cl).^32,73^ Bi_2_LaO_4_Cl, synthesized *via* the flux method, exhibits experimental optical band gap of 2.79 eV, in good agreement with our DFT-mBJ calculations, compared to 2.50 eV for Bi_2_YO_4_Cl and 3.16 eV for Bi_3_O_4_Cl.^74^ To further validate these results, the calculated optical properties of LaBi_2_ClO_4_ were compared with experimental trends for bismuth oxyhalides (BiOX, X = Cl, Br, I). Substituting Cl with Br or I narrows the band gap and shifts the absorption edge toward visible light, consistent with BiOX trends: BiOCl (3.44 eV), BiOBr (2.76 eV), BiOI (1.85 eV),^75^ and BiOBr_*x*_I_1−*x*_ solid solutions (2.87–1.89 eV).^76^ These effects arise from modified Bi–halogen hybridization and O 2p → Bi 6p transitions, corroborating experimental band edge shifts. The agreement confirms the reliability of our calculations and highlights halogen-doped LaBi_2_ClO_4_ as a promising visible-light optoelectronic and photocatalytic material.

## 4 Conclusions

First-principles DFT computations have been conducted to comprehensively examine the properties—including structural, mechanical, electronic, and optical aspects—of LaBi_2_ClO_4_ and its halogen-substituted forms LaBi_2_Cl_1–*y*_X_*y*_O_4_ (X = Br, I) at varying doping levels (*y* = 0.25, 0.5, 0.75). The negative formation energies confirm the thermodynamic stability of both the pristine and doped phases. Mechanical stability of the parent compound is validated by the satisfaction of the Born stability criteria. Analysis of the electronic structure indicates that pristine LaBi_2_ClO_4_ behaves as an indirect band gap semiconductor, with its band gap gradually narrowing as halogen atoms are substituted. Notably, iodine doping induces a stronger band gap reduction than bromine, attributed to greater lattice distortion. Optical properties display pronounced anisotropy, with halogen doping enhancing dielectric constants and refractive indices, while shifting absorption edges toward lower energies. These tunable optoelectronic properties highlight the potential of LaBi_2_ClO_4_ and its halogen-doped derivatives for application in next-generation optoelectronic devices.

## Author contributions

D. P. K. performed the DFT calculations focusing on the structural, electronic, and optical properties, and wrote the first draft of the manuscript in consultation with all co-authors. K. N. J. contributed to the DFT calculations related to thermodynamic stability and assisted in writing the manuscript, particularly the Introduction section. K. B. R. and S. L. carried out the calculations related to mechanical stability and properties. R. J. Y. and A. D. M. supervised part of the project, provide resources and analyzed the results. M. P. G. conceptualized and supervised the project, provided necessary resources, and directed the analysis in addition to writing the final version of the manuscript. All authors reviewed the content thoroughly and collectively endorsed the final manuscript for submission.

## Conflicts of interest

The authors report no conflicts of interest.

## Supplementary Material

RA-015-D5RA06049D-s001

## Data Availability

Most of the data that are used are kept within the manuscript and in the supplementary information (SI). The remaining data that support the findings of this research work will be made available from the corresponding author upon reasonable request. Supplementary information: formation energy, optimization, electronic band structures/DOS and related properties of LaBi_2_Cl_1−*y*_X_*y*_O_4_. See DOI: https://doi.org/10.1039/d5ra06049d.
