# Supplementary material for: Electronic and optical properties of halogen-substituted LaBi2Cl1−yXyO4: a promising candidate for energy-efficient devices
Source: RSC Adv. 2025 Sep 29;15(43):35930–40. doi: 10.1039/d5ra06049d (PMC12477683; doi:10.1039/d5ra06049d)
Supplement: RA-015-D5RA06049D-s001 [file RA-015-D5RA06049D-s001.pdf]

Supplementary Information (SI) for RSC Advances.
This journal is © The Royal Society of Chemistry 2025

Supplementary Information

Electronic and Optical Properties of Halogen-Substituted $LaBi_2Cl_{1-y}X_yO_4$: A Promising Candidate for Energy-Efficient Devices

Dipendra Prasad Kalauni[a], Kedar Nath Jaiswal[a], Sarita Lawaju[a], Krishna Bahadur Rai[a], Ram Jeewan Yadav[b], Akkal Dev Mishra[c], and Madhav Prasad Ghimire[a,d]

[a]Central Department of Physics, Tribhuvan University, Kirtipur 44613, Kathmandu, Nepal
[b]Department of Chemistry, Prithvi Narayan Campus, Pokhara, Tribhuvan University, Nepal
[c]Central Department of Chemistry, Tribhuvan University, Kirtipur 44613, Kathmandu, Nepal
[d]Leibniz IFW Dresden, Helmholtzstr. 20, 01069 Dresden, Germany

September 16, 2025

* Corresponding author: madhav.ghimire@cdp.tu.edu.np

Optimization

The Murnaghan equation of state is given by:

$$\mathrm{E(V)} = \mathrm{E_0} + \frac{\mathrm{BV}}{\mathrm{B'}}\left[\frac{1}{\mathrm{B'}-1}\left(\frac{\mathrm{V_0}}{\mathrm{V}}\right)^{\mathrm{B'}} + 1\right] - \frac{\mathrm{BV_0}}{\mathrm{B'}-1} \tag{S1}$$

The terms $\mathrm{E_0}$, V_0, B, and $\mathrm{B'}$ represent the equilibrium energy, the unit cell volume at zero pressure, the bulk modulus, and its first pressure derivative at P = 0, respectively.

Formation Energy

The formation energy (E_f) of $LaBi_2ClO_4$ and its halogen-substituted variants was calculated using the standard expression:

$$E_{\text{form}} = E_{\text{tot}} - aE^{\text{La}}_{\text{solid}} - bE^{\text{Bi}}_{\text{solid}} - cE^{\text{Cl}}_{\text{solid}} - dE^{\text{O}}_{\text{solid}}, \tag{S2}$$

For halogen-doped cases, the formation energy was computed as:

$$E_{\text{form}} = E_{\text{tot}} - aE^{\text{La}}_{\text{solid}} - bE^{\text{Bi}}_{\text{solid}} - cE^{\text{Cl}}_{\text{solid}} - dE^{\text{X}}_{\text{solid}} - fE^{\text{O}}_{\text{solid}}, \quad \text{X} = \text{Br, I}, \tag{S3}$$

where E_{tot} is the total energy of the fully relaxed unit cell, and a, b, c, d, and f denote the numbers of La, Bi, Cl, halogen (Br or I), and O atoms in the unit cell, respectively. $E^{\text{La}}_{\text{solid}}$, $E^{\text{Bi}}_{\text{solid}}$, $E^{\text{Cl}}_{\text{solid}}$, $E^{\text{X}}_{\text{solid}}$, and $E^{\text{O}}_{\text{solid}}$ represent the energies of the respective elements in their most stable solid phases. This methodology is widely employed in first-principles calculations to assess the thermodynamic stability of materials. For doped structures, formation energies were calculated by substituting the appropriate fraction of Cl atoms with Br or I, corresponding to doping levels of 25%, 50%, and 75%. The ground-state energies of the constituent elements were obtained using the crystal structures reported in the Materials Project database. These structures were fully optimized within our DFT framework prior to their use in the formation energy calculations. The crystal systems, space groups (SG), and optimized lattice parameters of each element in its stable phase are summarized below:

- **Bi**: Rhombohedral ($R\bar{3}m$), SG = 166, $a = b = 4.5380$ Å, $c = 12.3085$ Å, $\alpha = \beta = 90°$, $\gamma = 120°$.
- **La**: Hexagonal ($P6_3/mmc$), SG = 194, $a = b = 3.7963$ Å, $c = 12.0413$ Å, $\alpha = \beta = 90°$, $\gamma = 120°$.
- **Br**: Orthorhombic ($Cmca$), SG = 64, $a = 7.1395$ Å, $b = 4.2552$ Å, $c = 8.7454$ Å, $\alpha = \beta = \gamma = 90°$.
- **Cl**: Orthorhombic ($Cmca$), SG = 64, $a = 6.5284$ Å, $b = 4.1150$ Å, $c = 8.4024$ Å, $\alpha = \beta = \gamma = 90°$.
- **I**: Orthorhombic ($Cmca$), SG = 64, $a = 7.6792$ Å, $b = 4.6291$ Å, $c = 9.7962$ Å, $\alpha = \beta = \gamma = 90°$.
- **O**: Monoclinic ($C12/m1$, C-base centered), SG = 12, $a = 8.7549$ Å, $b = 6.2202$ Å, $c = 4.3898$ Å, $\alpha = \gamma = 90°$, $\beta = 115.7669°$.

These optimized structural parameters provide the basis for accurate ground-state energy evaluation of the constituent elements, which is essential for obtaining reliable formation energies of the studied compounds.

Electronic and Optical properties

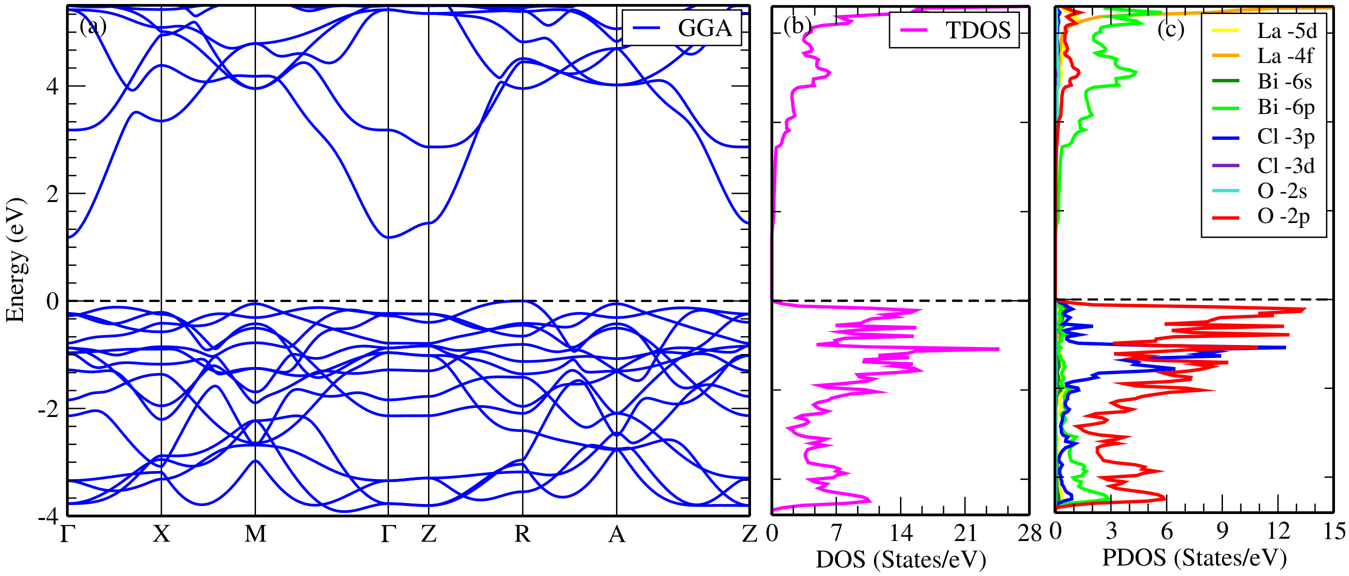

Figure S1: (a) Electronic band structure, (b) total density of states (DOS), and (c) projected density of states (PDOS) of $LaBi_2ClO_4$ calculated using the GGA potential. The horizontal dashed line indicates the Fermi level (E_F).

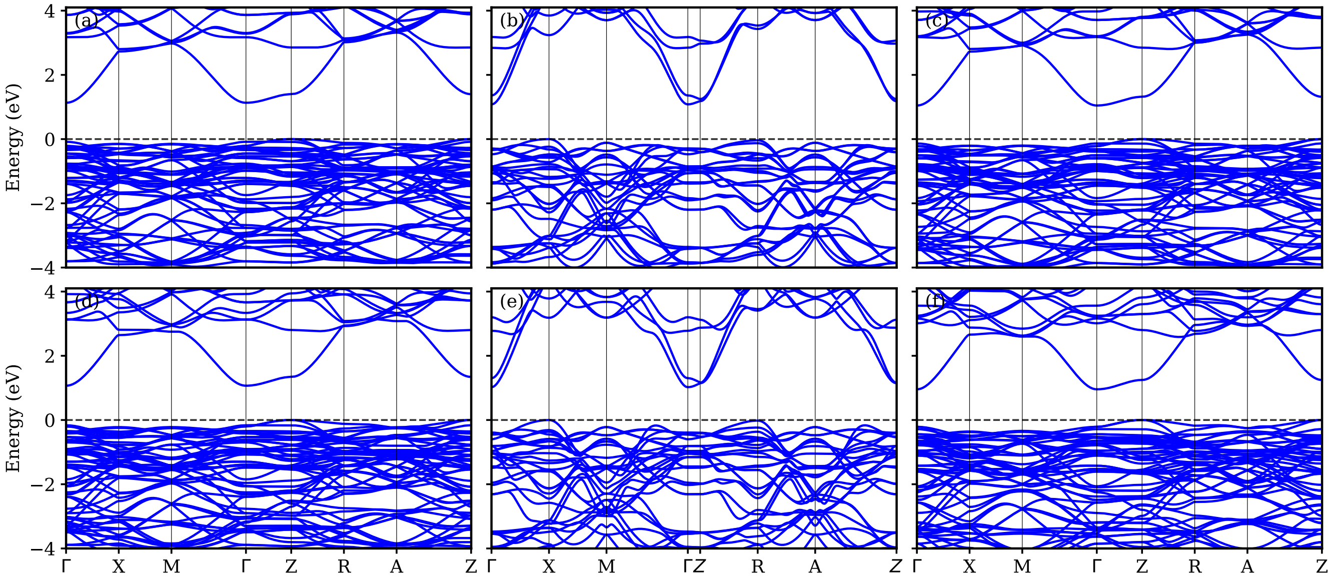

Figure S2: Electronic band structures of $LaBi_2ClO_4$ with partial substitution of Cl by Br and I at various doping concentrations, calculated using the GGA potential: (a) $LaBi_2Cl_{0.75}Br_{0.25}O_4$, (b) $LaBi_2Cl_{0.5}Br_{0.5}O_4$, (c) $LaBi_2Cl_{0.25}Br_{0.75}O_4$, (d) $LaBi_2Cl_{0.75}I_{0.25}O_4$, (e) $LaBi_2Cl_{0.5}I_{0.5}O_4$, and (f) $LaBi_2Cl_{0.25}I_{0.75}O_4$.

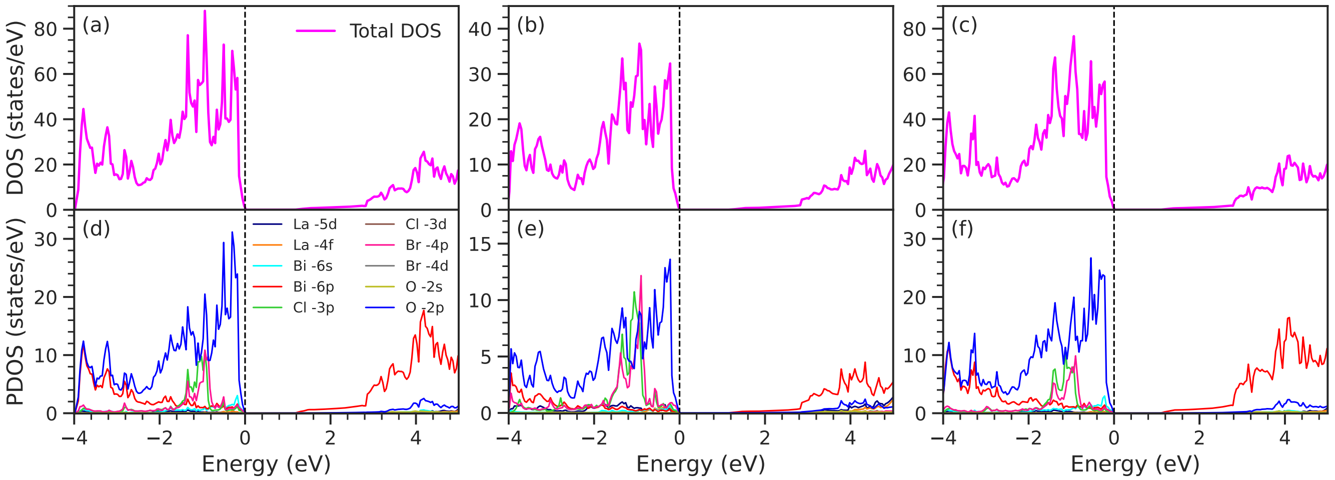

Figure S3: Calculated total density of states (DOS) and partial density of states (PDOS) of $LaBiCl_{1-y}Br_yO_4$ for doping levels $y = 0.25$, 0.50, and 0.75, computed using the GGA functional. Panels (a), (b), and (c) show the total DOS for 25%, 50%, and 75% Br substitution, respectively, while panels (d), (e), and (f) present the corresponding PDOS for these doping concentrations.

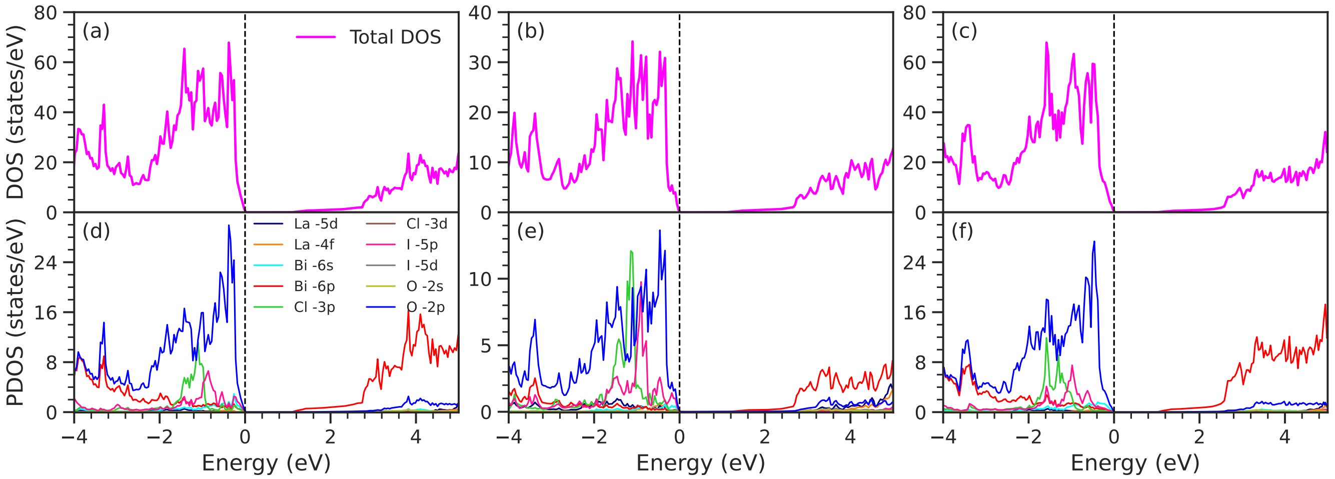

Figure S4: Calculated total density of states (DOS) and partial density of states (PDOS) of $LaBiCl_{1-y}I_yO_4$ for doping levels $y = 0.25$, 0.50, and 0.75, computed using the GGA functional. Panels (a), (b), and (c) show the total DOS for 25%, 50%, and 75% Br substitution, respectively, while panels (d), (e), and (f) present the corresponding PDOS for these doping concentrations.

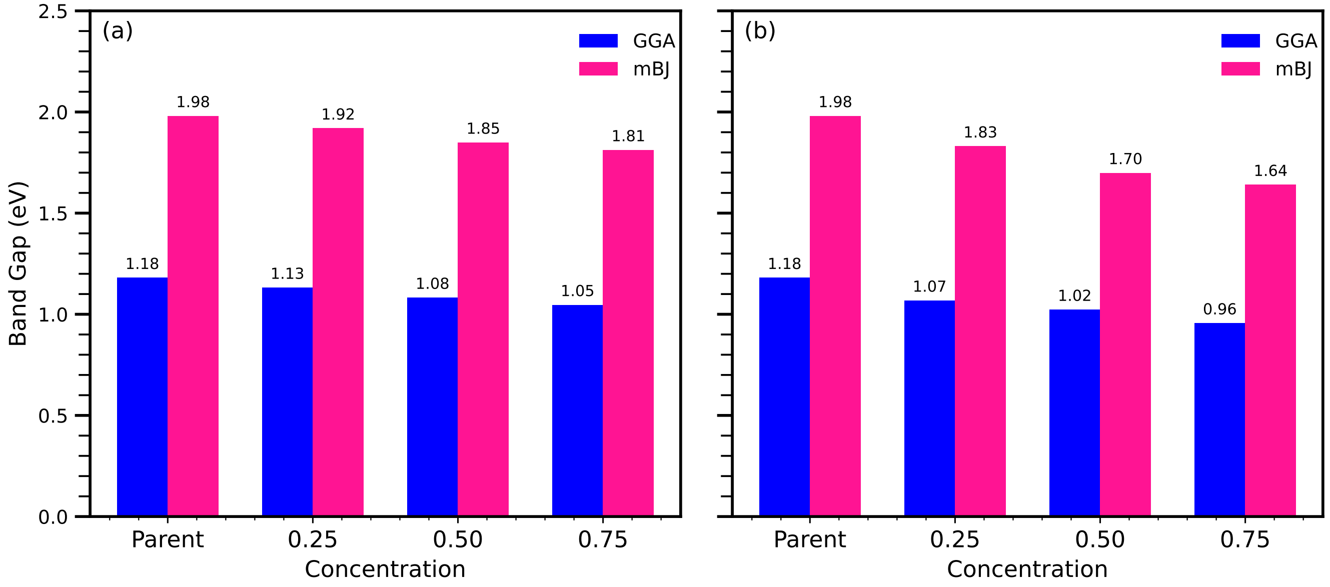

Figure S5: (a) Variation of the electronic band gap (in eV) with Br substitution at the Cl site in $LaBi_2ClO_4$ ($LaBi_2Cl_{1-y}Br_yO_4$), calculated using both the GGA and mBJ exchange-correlation approaches. (b) Corresponding variation of the band gap with I substitution at the Cl site in $LaBi_2ClO_4$ ($LaBi_2Cl_{1-y}I_yO_4$). In both cases, a gradual reduction in the band gap is observed with increasing halogen concentration, indicating a tunable electronic structure via halogen doping.

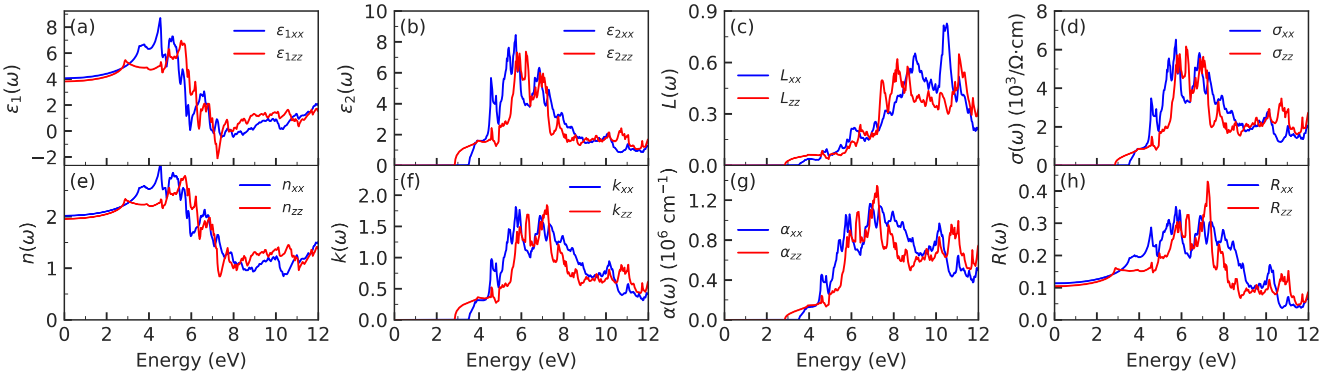

Figure S6: Calculated optical properties of $LaBi_2ClO_4$ as functions of photon energy using the GGA exchange potential. Panels show: (a) real $\varepsilon_1(\omega)$ and (b) imaginary $\varepsilon_2(\omega)$ parts of the dielectric function; (c) energy loss function $L(\omega)$; (d) optical conductivity $\sigma(\omega)$; (e) refractive index $n(\omega)$; (f) extinction coefficient $k(\omega)$; (g) absorption coefficient $\alpha(\omega)$; and (h) reflectivity $R(\omega)$. The subscripts xx and zz denote the polarization directions along the crystallographic x- and z-axes, respectively.

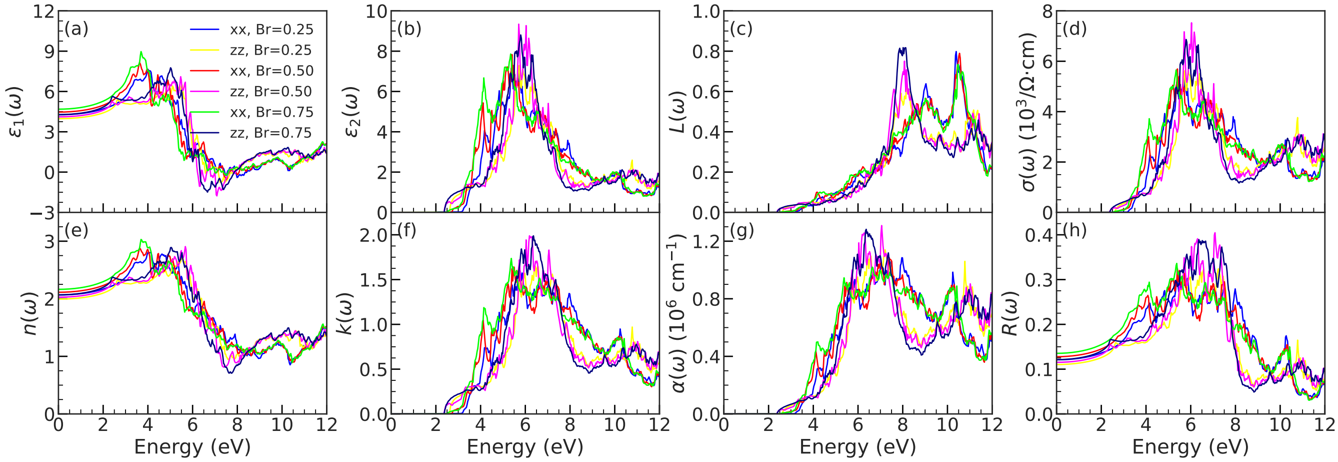

Figure S7: Calculated optical properties of $LaBi_2Cl_{1-y}Br_yO_4$ for $y = 0.25$, 0.50, and 0.75 as functions of photon energy, computed using the GGA exchange-correlation potential. Panels show: (a) real part of the dielectric function, $\varepsilon_1(\omega)$; (b) imaginary part, $\varepsilon_2(\omega)$; (c) energy loss function, $L(\omega)$; (d) optical conductivity, $\sigma(\omega)$; (e) refractive index, $n(\omega)$; (f) extinction coefficient, $k(\omega)$; (g) absorption coefficient, $\alpha(\omega)$; and (h) reflectivity, $R(\omega)$. The subscripts xx and zz represent polarization directions along the crystallographic x- and z-axes, respectively.

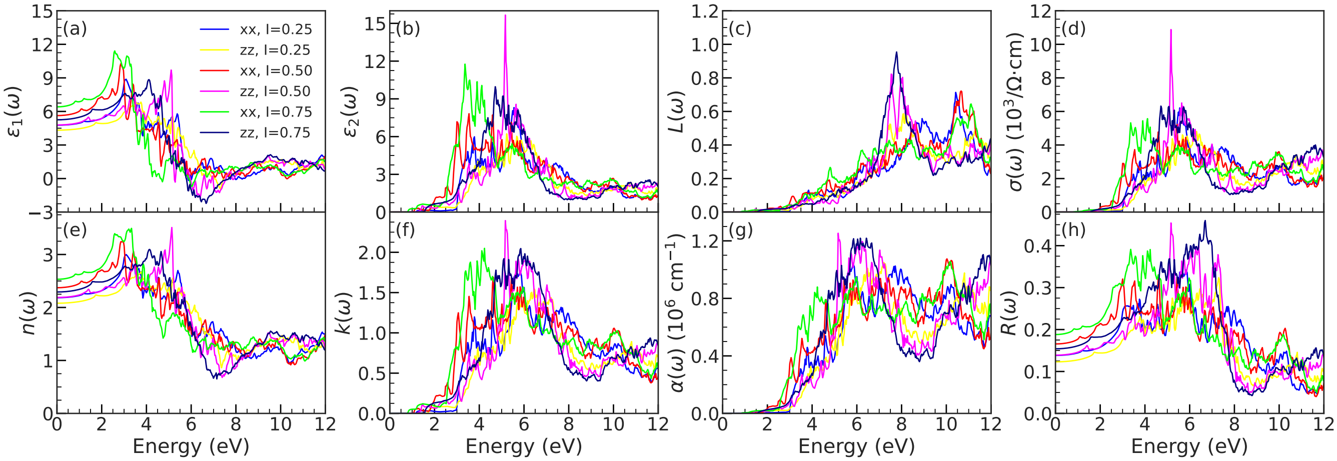

Figure S8: Calculated optical properties of $\mathrm{LaBi_2Cl_{1-y}I_yO_4}$ for $y = 0.25$, 0.50, and 0.75 as functions of photon energy, computed using the GGA exchange-correlation potential. Panels show: (a) real part of the dielectric function, $\varepsilon_1(\omega)$; (b) imaginary part, $\varepsilon_2(\omega)$; (c) energy loss function, $L(\omega)$; (d) optical conductivity, $\sigma(\omega)$; (e) refractive index, $n(\omega)$; (f) extinction coefficient, $k(\omega)$; (g) absorption coefficient, $\alpha(\omega)$; and (h) reflectivity, $R(\omega)$. The subscripts xx and zz represent polarization directions along the crystallographic x- and z-axes, respectively.
